# Supplementary material for: Association between serum uric acid and spirometric pulmonary function in Korean adults: The 2016 Korea National Health and Nutrition Examination Survey
Source: PLoS One. 2020 Oct 22;15(10):e0240987. doi: 10.1371/journal.pone.0240987 (PMC7580900; doi:10.1371/journal.pone.0240987)
Supplement: Attachment — Submitted filename: reply to reviewer 20201005.docx [file pone.0240987.s002.docx]

Reply to Reviewers

Reviewer #3: The sentence of below can be misled readers without references. I strongly recommend to update references. If not, it is needed to be deleted.
"Women may have greater xanthium oxidase activity and reactive
oxygen species (ROS) production, resulting in severe vascular inflammation, arterial stiffness,
and elevated blood pressure as compared with men."

- Thank you for your comments. As your suggestion, we deleted those sentences to avoid unnecessary misunderstanding
